# The inequality of inpatient care net benefit under integration of urban-rural medical insurance systems in China

**DOI:** 10.1186/s12939-018-0891-0

**Published:** 2018-11-22

**Authors:** Xue Yang, Mingsheng Chen, Jinglin Du, Zhonghua Wang

**Affiliations:** 1 School of Health Policy & Management, Nanjing Medical University, 101Longmian Avenue, Jiangning District, Nanjing, 211166 People’s Republic of China; 2 Creative Health Policy Research Group, Nanjing Medical University, Nanjing, 211166 China

**Keywords:** Health insurance system, Benefit distribution, Inequality, Concentration index

## Abstract

**Background:**

China has recently made efforts to integrate urban and rural basic medical insurance systems in order to ensure both urban and rural enrollees obtain unified benefits. However, whether the distribution of government healthcare subsides has become more equitable remains unknown. The purpose of this study was to analyze determinants of and inequality in net inpatient care benefits under the integration of urban-rural medical insurance systems in China.

**Methods:**

Data were obtained from a nationally representative household survey, the Fifth National Health Services Survey (2013), conducted in Anhui province. A multiple regression model and concentration index (CI) was used to estimate related factors and inequality of inpatient care net benefits.

**Results:**

Findings indicated that individuals received more inpatient care benefits when urban and rural social healthcare systems were integrated. Factors associated with net benefits included gender, age, marital status, retirement, educational level, history of chronic diseases, health status, willingness to seek inpatient care and per capita income. The rich were found to disproportionately benefit from inpatient care, and the CI of net benefits for integrated insurance enrollees was the lowest among all three available health insurance schemes. These findings indicate that the recent unification of urban-rural social health insurances reduces inequality in net benefits from government subsidies. Some socioeconomic factors, such as per capita income, 60 years of age and over, history of chronic disease and high educational level positively influence inequality.

**Conclusion:**

In China, accelerating the integration of urban and rural medical insurance systems is an effective way to increase equity of benefit in urban and rural areas. Strategies aimed at reducing inpatient benefit inequality must address socioeconomic factors influencing healthcare outcomes.

## Introduction

Achieving universal health coverage(UHC) which provides all individuals and communities with quality health services they need while sparing financial hardship, is one of the important targets of the Sustainable Development Goals set forth by the United Nations in 2015 [1]. The ultimate goal of social health insurance is to provide affordable, cost-effective, and equitable healthcare for all [2]. Since 2009, the Chinese government has been deepening the health care system reform with the goal of ultimately providing affordable and equitable basic health services for citizens [3]. As the dominant target of reform measures, the social health insurance system initially implemented strategies aimed at achieving basic universal coverage and steadily expanded benefits over the last decade [3]. Mainly three programs compose the Chinese social health insurance system. These include the Urban Employee Basic Medical Insurance (UEBMI) established in 1998 for individuals employed in the urban sector, the New Rural Cooperative Medical Scheme (NRCMS) launched in 2003 to cover the large rural population, and the Urban Resident Basic Medical Insurance (URBMI) implemented in 2007 to cover urban residents without formal employment. By 2018, more than 1.35 billion people enrolled in these three programs, equivalent to 95% of the total population [4].

While China’s basic medical insurance has made great achievements, its framework highlights a few inherent structural problems. The long-standing urban-rural split in social health insurance systems, which not only hinders urban-rural integration but also causes inequality in government subsidies, is a major issue yet to be resolved. China has been making efforts to integrate URBMI and NRCMS thereby providing urban and rural residents with a unified coverage range, financing mechanism, benefits package, and fund management since 2012 [5]. This program was called the Urban and Rural Residents’ Basic Medical Insurance System (URRBMI) [6]. The key objective of the URRBMI is to ensure that all participants obtain equal and comprehensive access to healthcare benefit packages, irrespective of financial status or living location. By the end of March 2015, 8 provinces, 39 cities and over 100 counties touted successful urban and rural integration of the basic medical insurance system [7]. URRBMI enrollees exceeded 0.87 billion by the end of 2017, and the preliminary framework for a unified urban-rural medical insurance was successfully formed [8]. Although the system has achieved resource and institutional integration, cost reduction and the narrowing of healthcare resource allocation gaps between urban and rural areas [7], whether the benefit distribution of government healthcare subsides across different socioeconomic groups has become more equitable remains unknown.

Some studies have reported that expansion of health insurance coverage increases access to benefits and healthcare utilization among the poor [9, 10]. Extending insurance coverage is thus considered to contribute to greater financial risk protection [11]. However, in most developing countries, distribution of government healthcare subsidies favored the rich population [12, 13]. Moreover, health insurance programs funded by different mechanisms may result in differing patterns of benefit distribution. Huang et al. reported that government health subsidy benefiting the poor more than the rich was observed among those who paid wage-based premiums, with the lowest income group receiving the highest net benefits and the richest income group receiving the lowest. However, there is less evidence for such a vertically equitable pattern in people who paid fixed premiums [14]. Several studies have also evaluated the causes of inequality in government healthcare subsidies and benefits distribution. Makawia and Borghi reported that because of differences in benefit packages, members of the National Health Insurance Fund (NHIF) received more services and benefits than did enrollees of the Community Health Fund (CHF) [9]. In contrast to China’s universal coverage of social health insurances, NHIF targeting at the people from formal sector with a wider range of benefits at all levels of facilities covers 7.2% of the population, while CHF for populations in the rural areas and the informal sector with the benefits limited in public primary health facilities covers about 6.6% population in Tanzania [15, 16]. If the health insurance scheme only reaches a small proportion of the population then it will be difficult for it to impact on improving equity of access for the health system more generally [17]. Lu and Hsiao found that misdistribution of healthcare resources adversely influenced receipt of government subsidies and benefits by poorer people or those living in remote areas [11]. Macha et al. explored the factors influencing the distribution of healthcare benefits from the perspective of availability, affordability and acceptability, and found that high cost for seeking health care, limited access to higher-level facilities among poor and rural populations and staff attitudes were the main factors leading to distribution of healthcare benefits in Ghana, Tanzania and South Africa that disproportionately benefited the rich [18].

As China faces dramatic increases in healthcare costs, the social health insurance fund plays an increasingly significant role in reducing the financial barrier to healthcare access for all citizens [19]. As health insurance programs are mainly funded by government revenue, the distribution of healthcare benefits among NRCMS or URBMI enrollees has drawn a great deal of public policy researchers attention over the last decade. Some studies have focused on evaluating NRCMS or URBMI programs exclusively. Wang et al. defined a net benefit by subtracting the premium and co-payment from the value of services received by a patient [20]. Pan et al. considered the absolute amount of reimbursement received to represent the net benefit, better capturing the essence of equity than focusing on relative measures [21]. Wang et al. (2005) and Meng et al.(2008) reported that richer participants benefited more from government healthcare subsidies among people enrolled in NRCMS with income and health status being major factors influencing net benefit [20, 22]. Pan et al. (2016) reported that beneficiaries from lower income groups benefited less than those from higher income groups among URBMI enrollees. Also, income and age were the main influencing factors of health expense reimbursement [21].

Although the Chinese health insurance system for urban and rural residents has been established for over 10 years and several prior studies have examined NRCMS or URBMI, few studies have focused on the integration of the new urban-rural medical insurance system. Furthermore, to our knowledge, there has been no research on the analysis of inequality in government healthcare subsidy distribution among people enrolled in URRBMI in China. Therefore, the aim of the present study was to analyze determinants and inequalities in net benefits of inpatient care under the integrated Chinese urban-rural insurance system and compare outcomes of URBMI, NRCMS and URRBMI enrollees. Using Fifth National Health Service Survey data, we estimated the impact related socioeconomic factors exerted on net benefits of inpatient care received under the urban-rural scheme. We then applied the concentration index (CI) and its decomposition to measure inequalities in the net benefits of inpatient care and estimate influences individual factors exerted on inequality.

## Method

### Data source

This study used data from the Fifth National Health Services Survey (NHSS) in Anhui province conducted in 2013. NHSS was conducted by the Statistics Information Center of the Ministry of Public Health, adopting the multi-phase stratified cluster random sampling method, and aimed to construct a high-quality nationally representative sample of the Chinese population. The NHSS has been conducted every 5 years and the 2013 survey was the fifth such survey. As the designated investigation team, we conducted the related survey in Anhui province. It should be noted that the 2013 survey data is the latest data of NHSS available at present. Anhui province, located in east China, is a major agricultural province with a large population [23]. Data from Anhui province used in this study included eight county-level units^1^ (four counties and four municipal districts). Each county-level unit randomly sampled five towns or streets. Two village-level units (two communities or two villages) were subsequently selected from each town-level unit. Household units, as the final sampling units, were extracted from village-level units by a system sampling method, and 60 households were selected from each village or community. The household survey eventually included approximately 4800 sampled households and 13,986 participants aged≥15. Among them, the total of people with NRCMS, URBMI or URRBMI is 11,468. Respondents who had any missing variables were excluded to ensure an accurate analysis. A total number of 9258(66%) participants were enrolled in our study. Among individuals included, 5191 were enrolled in NRCMS, 1345 were enrolled in URBMI and 2722 were enrolled in URRBMI.

NHSS data provides detailed information concerning individuals’ demographic characteristics, economic status, health status, healthcare utilization and costs, healthcare insurance status, distance to medical facilities and willingness to seek medical services. Information about all subjects was collected by household questionnaires and each eligible member of selected households was interviewed by the trained interviewers. Statistical analyses were performed with STATA 14.0.

### Data analysis

#### Variable selection

In our analyses, reimbursement received from NRCMS, URBMI or URRBMI was considered a measure of net benefit. The reimbursement ratio or reimbursement amount reflected the actual use of social health insurances, namely, for purposes of studying the distribution of insurance benefits from the perspective of result fairness [24]. The dependent variable “net benefit” was rooted from subsidies the governments contribute to beneficiaries who participated in social health insurance schemes and actually utilized inpatient services, in agreement with Yuan S et al. reporting that the enrollees realize the indeed benefit from government subsidy according to the arrangement of the reimbursement procedure [25] and also Pan et al. defined net benefit as the amount of reimbursement and indicated that the net benefits come from the subsidies the governments contribute to each participant [21]. Social health insurances in our study are mainly financed by government subsidy and those who utilize more services provided by health providers reimbursed by NRCMS, URBMI and URRBMI will receive more benefits [20, 21, 25]. Hence, essentially, net benefit or the amount of reimbursement received from social health insurances mainly comes from government subsidies. In this study, we focused only on evaluating the integration of the urban-rural insurances system (NRCMS, URBMI and URRBMI). NRCMS, URBMI and URRBMI are mainly funded by government revenue and the insurers paid relatively fixed premiums, allowing us to accurately measure net healthcare benefits provided. Therefore, we used the absolute amount of reimbursement received as a measure of net benefit (NB) by subtracting out-of-pocket (OOP) expenses from the total cost of hospitalization.

Based on related literatures, independent variables concerning demographic characteristics included age, gender, marital status, educational level and employment status [21]. Economic status was reflected by per capita income [26]. Health status factors included chronic diseases and self-reported health status. Chronic disease status was divided into two groups (1.chronic diseases, 2. non-chronic disease). Self-reported health status was measured by the Visual analogue scale (VAS) in the questionnaire and was divided into five grades in accordance with previous studies conducted by JIA et al. and Wang et al. [27, 28]. Distance to the medical institution was classified as either less than one kilometer or one kilometer and above. Willingness to seek medical services was categorized into two dimensions (1.primary hospital, 2. general hospital or traditional Chinese medicine hospital) and was used to reflect participants’ health-seeking behavior. Table 1 presents details dependent and independent variables.

**Table 1:** Description of dependent and independent variables

	Description	Indicators/survey questions
Dependent variable		
Net benefit of inpatient care	the absolute amount of reimbursement received(yuan RMB)	Subtract individual payment from the total hospitalization expense
Independent variables		
Gender	=1 if male; =2 if female.	
Age	=1 if≤45; =2 if 45–59; =3 if ≥60.	
Marital status	=1 if married; =2 if divorced/single/separated/other.	Question: What is your present marital status?
Educational level	=1 if under primary school; =2 if junior; =3 if high school or above.	The highest degree of education received or a degree equivalent to an existing cultural level
Chronic	=1 if no chronic; =2 if one or multiple chronic.	Question: Have you been diagnosed with any chronic diseases by a doctor? Chronic diseases listed subsequently.
Distance to the medical institution	=1 if < 1 km; =2 if≥1 km.	Question: How many kilometers is the nearest medical institution from your home?
Willingness to seek medical services	=1 if primary hospital(village/private clinics/community health service station/township hospital/community health service center); =2 if general hospital or TCM hospital (traditional Chinese medicine)	Question: For general diseases, what kind of medical institutions do you and your family members go to?
Health status (EQ-VAS: self-reported)	=1 if very poor(0–20); =2 if poor(21–40); =3 if medium(41–60); =4 if good(41–80); =0 if excellent(81–100).	Health status on a 20 cm vertical scale with end points of 0 and 100 was asked on the day of the interview.
Employment status	=1 if employment; =2 if retirement; =3 if student or unemployment.	Question: What is your present employment situation? [conditions listed below, read one by one]
Per capita income	Household per capita income over the last year(yuan RMB)	

#### Multiple regression models on net benefit of inpatient care

Multiple regression was applied to identify determinants of net benefit. Here, we define x_k_ as factors related to NB. The multiple linear regression model was defined as follows:

1 $$ {Y}_i=\alpha +{\sum}_k{\beta}_k{\chi}_{ki}+{\varepsilon}_i $$

where Y represents NB (the log value of the absolute amount of reimbursement) of inpatient care. x_k_ denotes demographic characteristics, economic status and other related factors, wherein per capita income is also denoted with log values to avoid data nonstationarity.

#### Concentration index and its decomposition in net benefit of inpatient care

In order to reflect net benefit inequality of inpatient care, we used a concentration index (CI) which is commonly used to measure socioeconomic-related health inequality [29–31]. The concentration index (CI) quantifies the degree of socioeconomic-related inequality in a health or healthcare variable and is defined as twice the area between the concentration curve and the line of equality. The concentration curve plots shares of the health variable against quantiles of the living standards variable [29]. In this study, the distribution of net benefit of inpatient care is examined by income quintiles, beginning with the poorest and ending with richest:

2 $$ CI= 2/\mu\;\mathit{\operatorname{cov}}\left(h,r\right) $$

Where *h* denotes the healthcare sector variable; *μ* is its mean; and *r* is the fractional rank of individuals in distribution of economic status. Here, CI is defined as the distribution of net benefit of inpatient care contributions across the population as ranked by per capita income. Individuals are sorting in ascending order of per capita income and are grouped into five income quintiles. The CI is positive if the net benefit distribution is progressive, suggesting that the rich receive more reimbursements than the poor. The value of CI is negative if the distribution of net benefit is regressive. And the value is zero, implying the absolute fairness. Furthermore, if the positive value is larger, the pro-rich pattern is more significant. If the absolute value of negative CI is higher, it means that the pro-poor pattern is more significant.

Concentration index may be decomposed into individual factors contributions to net benefit inequality, where each contribution is the product of the sensitivity of net benefit with respect to that factor and the degree of inequality in that factor [29]. Furthermore, the corresponding contribution rate is a measure of the proportion of its income-related inequality to CI. The decomposition of CI can be calculated as follows:

3 $$ CI={\sum}_k\left({\beta}_k{\overline{\chi}}_k/\overline{Y}\right){CI}_k+{GCI}_{\varepsilon }/\overline{Y} $$

4 $$ Contribution\kern0.17em rate={CI}_k/ CI $$

where $\overline{\mathrm{Y}}$ is the mean net benefit; *β*_*k*_ is the coefficient of multivariate linear regression in eq. 1; ${\overline{\mathrm{X}}}_{\mathrm{k}}$ is the mean x_k_; CI_k_ is the concentration index for x_k_; and GCI _ε_ is the generalized concentration index of the error term ε.

## Results

Table 2 presents descriptive statistics of dependent and independent variables by type of medical insurance. As shown in Table 2, residents enrolled in URRBMI, NRCMS and URBMI received 338, 285, and 229 Yuan from government subsidies, respectively. Among individuals included in the survey, nearly 50% were male, except for individuals enrolled in URBMI (38% were male and 62% were female). In all insurance schemes, more than 42% were aged ≤44 and more than 22% were aged over 60. In addition, 76, 45 and 61% of respondents enrolled in NRCMS, URBMI and URRBMI were employed, respectively. Individuals enrolled in URBMI demonstrated a significantly higher proportion of at least a high school education level (35%) than did respondents enrolled in NRCMS (10%) or URRBMI (12%). 57% of people enrolled in URRBMI were found to have a low level of education (less than primary school). 7% of individuals enrolled in NRCMS or URRBMI and 9% of people enrolled in URBMI suffered from one or multiple chronic diseases. 72% of URBMI enrollees could access health facilities within 1 kilometer of their residence, a significantly greater percentage than individuals enrolled in NRCMS (47%) or URRBMI (43%). Nearly 95% of respondents enrolled in NRCMS or URRBMI were willing to attend a primary care hospital for general diseases when compared to 73% of URBMI enrollees. Per capita income was highest (14,473 Yuan) among individuals enrolled in URRBMI (14,473 Yuan) and lowest among NRCMS enrollees (12,451 Yuan).

**Table 2:** Sample data

Variables	NRCMS	URBMI	URRBMI	sign
	Mean (S.D.)	Mean (S.D.)	Mean (S.D.)	
Dependent variable				
Net benefit of inpatient care	229.0460 (2106.3960)	285.0119 (1953.7150)	338.0393 (3185.9190)	
Independent variable				
Gender				***
Male	0.5005 (0.5)	0.3799 (0.4855)	0.4699 (0.4992)	
Female	0.4995 (0.5)	0.6201 (0.4855)	0.5301 (0.4992)	
Age				***
≤ 44	0.4879 (0.4999)	0.5219 (0.4997)	0.4232 (0.4942)	
45–59	0.2791 (0.4486)	0.2506 (0.4335)	0.2946 (0.456)	
≥ 60	0.233 (0.4228)	0.2275 (0.4194)	0.2821 (0.4501)	
Marital status				***
Married	0.791 (0.4066)	0.6669 (0.4715)	0.7855 (0.4106)	
Divorced/single/separated/other	0.209 (0.4066)	0.3331 (0.4715)	0.2145 (0.4106)	
Employment/retirement status				***
Employment	0.757 (0.4289)	0.4528 (0.498)	0.6058 (0.4888)	
Retirement	0.0085 (0.0918)	0.0862 (0.2808)	0.0121 (0.1095)	
Unemployment or student	0.2345 (0.4237)	0.461 (0.4987)	0.3821 (0.486)	
Educational level				***
Under primary school	0.4962 (0.5)	0.3011 (0.4589)	0.5665 (0.4956)	
Junior	0.3997 (0.4899)	0.3517 (0.4777)	0.3108 (0.4629)	
High school or above	0.1041 (0.3054)	0.3472 (0.4763)	0.1227 (0.3282)	
Chronic				*
No chronic	0.9312 (0.2531)	0.913 (0.2819)	0.9342 (0.2479)	
One or multiple chronic	0.0688 (0.2531)	0.087 (0.2819)	0.0658 (0.2479)	
Health status (EQ-VAS: self-reported)				***
Excellent	0.5528 (0.4972)	0.5591 (0.4967)	0.6025 (0.4895)	
Good	0.3463 (0.4758)	0.345 (0.4755)	0.3185 (0.466)	
Medium	0.0887 (0.2843)	0.0855 (0.2797)	0.0632 (0.2433)	
Poor	0.0104 (0.1016)	0.0089 (0.0941)	0.0121 (0.1095)	
Very poor	0.0017 (0.0417)	0.0015 (0.0385)	0.0037 (0.0605)	
Distance to the medical institution				***
< 1 km	0.4742 (0.4994)	0.7182 (0.45)	0.4335 (0.4956)	
≥ 1 km	0.5258 (0.4994)	0.2818 (0.45)	0.5665 (0.4956)	
Willingness to seek inpatient care				
Primary care hospital	0.9581 (0.2004)	0.7331 (0.4425)	0.9556 (0.2061)	***
General hospital or TCM hospital (Traditional Chinese Medicine)	0.0419 (0.1972)	0.2669 (0.4467)	0.0444 (0.271)	
Per capita income	12,450.55 (13,065.36)	13,735.92 (10,404.71)	14,473.30 (18,066.56)	***

Note: Primary care hospital refers to village / private clinics, community health service stations, township hospitals or community health service centers; general hospital refers to general hospitals at the municipal / county level; TCM hospital refers to traditional Chinese medicine hospitals at the municipal /county level. Calculations were weighted using individual sampling weights and adjusted for individual responses

***implies *p*-value < 0.001, *implies *p*-value< 0.05

Table 3 shows regression results of factors that influenced inpatient benefit. As indicated in Table 3, females and those aged 60 years and over demonstrated a positive association with benefits of inpatient care received by all beneficiaries, implying that females and the elderly received more benefits than did males or those aged 44 years and under. This effect was estimated to be greatest in NRCMS enrollees. Participants who were divorced, single or separated received significantly less benefits in comparison with married individuals; such an effect was also estimated to be greater among those enrolled in NRCMS. Compared with employed individuals, the retired received significantly more net benefits of inpatient care among URBMI enrollees. In addition, there was a positive association between educational levels and net benefits of inpatient care received in all health insurance types. Results revealed that enrollees with chronic diseases had greater reimbursement in comparison with those without chronic disease in all insurance schemes, and this effect was estimated to be higher in URRBMI enrollees. Individuals who self-rated their health as poor and very poor received significantly higher net benefits in comparison with those who reported excellent health, especially individuals enrolled in URBMI, who exhibited the highest effect of health status on net inpatient care benefits received. Individuals or their relatives who were willing to go to a general or traditional Chinese medicine (TCM) hospital for general diseases also received greater benefits when compared with those who were willing to visit a primary care hospital. Per capita income was positively correlated with net benefits received; this effect was estimated to be greater in NRCMS enrollees.

**Table 3:** Multiple regression model analysis of net benefit of inpatient care by types of medical insurance schemes for urban and rural residents

	NRCMS		URBMI		URRBMI	
	Coef.	SD	Coef.	SD	Coef.	SD
Gender, ref.: Male	0.21**	0.09	0.09***	0.01	0.06*	0.04
Age, ref.:≤44						
45–59	0.07	0.11	−0.08	0.14	−0.03*	0.02
≥ 60	0.31***	0.04	0.18**	0.09	0.17*	0.09
Marital status, ref.: married	−0.41***	0.08	−0.29***	0.06	−0.17***	0.02
Employment/retirement status, ref.: employment						
Retirement	0.44	0.46	0.28**	0.14	−0.31	0.29
Unemployment or student	−0.13	0.16	−0.08	0.07	−0.21	0.24
Educational level, ref.: Under primary school						
Junior	0.11	0.14	0.17**	0.08	0.18**	0.09
High school or above	0.34***	0.07	0.48**	0.23	0.44***	0.11
Chronic, ref.: No chronic	1.28***	0.04	1.34***	0.43	1.59***	0.31
Health status(EQ-VAS: self-reported), ref.: Excellent						
Good	0.23	0.35	0.43	0.51	0.37	0.31
Medium	0.77	0.68	0.92*	0.48	0.65	0.57
Poor	1.26***	0.14	1.61***	0.30	1.19***	0.21
Very poor	1.41**	0.70	1.84***	0.19	1.32**	0.62
Distance to the medical institution, ref.:< 1 km	0.03*	0.02	−0.04	0.06	−0.07	0.11
Willingness to seek inpatient care, ref: Primary care hospital						
General hospital or TCM hospital (Traditional Chinese Medicine)	0.33**	0.15	0.29***	0.07	0.41***	0.08
Per capita income	0.09***	0.01	0.03**	0.01	0.02**	0.01

Note: Estimates were weighted using individual sampling weights and adjusted for individual responses

*** implies *p*-value < 0.01, ** indicates *p*-value < 0.05, * implies *p*-value < 0.1

Table 4 shows the CIs of inpatient benefits available under medical insurance schemes for urban and rural residents and the mean per capita income of five quintiles. There were considerable differences in per capita income across the income groups among enrollees of all insurance schemes. Furthermore, the mean value of the richest group was approximately eight times that of the poorest group. The CI of benefit distributions among individuals enrolled in NRCMS, URBMI and URRBMI were all positive, indicating that a larger proportion of benefits was delivered to the rich than to the poor. However, the absolute value of CI of NRCMS enrollees was greatest (0.2473), while that of URRBMI enrollees was lowest (0.1032). These findings suggest that inequalities favoring the rich were mitigated under the integration of urban and rural insurance schemes.

**Table 4:** Concentration indexes of inpatient benefit by types of medical insurance schemes for urban and rural residents

	NRCMS		URBMI		URRBMI	
	Mean	SD	Mean	SD	Mean	SD
Poorest	3477.23	1245.09	4054.81	1536.99	4552.67	1772.40
Poorer	6420.31	836.93	7955.92	1106.20	8570.83	1090.02
Middle	9453.21	790.61	11,070.22	1124.70	11,256.51	1183.34
Richer	14,003.37	1787.52	16,057.94	1640.87	15,737.60	1998.51
Richest	28,885.67	21,188.57	29,446.30	12,098.19	32,222.20	34,089.13
Concentration index	0.2473		0.1618		0.1032	

Note: Calculations were weighted using individual sampling weights and adjusted for individual responses

Table 5 to Table 7 present contributions of each factor associated with the inequality of net inpatient care benefits received of respective schemes. As shown in the last column of Table 5, the total contribution percentage was 102.8%, implying that 2.8% of the negative contribution to inequality was explained by the error term of the regression. The results of the last column of Table 6 and Table 7 are on the analogy of this. A positive contribution to inequality implied that relevant variable increased inequality, and vice versa. As shown in Table 5, with the exception of marital and self-reported health statues, contributions of other factors were all positive. The majority of observed inequalities (0.2473) in net inpatient care benefit among individuals enrolled in NRCMS was positively attributed to per capita income (40%), age ≥ 60 (21%) and suffering chronic diseases (13%). As displayed in Table 6, among individuals enrolled in URBMI, the majority of observed inequities (0.1618) in net benefits were attributed to age ≥ 60 (31.95%), high level of education (28.87%), and per capita income (11.49%). Other factors, such as poor self-reported health status and distance to a medical institution of ≥1 km, had slightly negative contributions to inequality. As shown in Table 7, in URRBMI, Age ≥ 60 (30%), a high educational level (22%), per capita income (19%), history of chronic diseases (18%) and attending a general or TCM hospital for medical services (14%) were the major positive contributors of net benefit inequality. Health status and retirement had slightly negative contributions to inequality.

**Table 5:** Decomposition of concentration index of inpatient benefits among New Rural Cooperative Medical Scheme (NRCMS) enrollees

	the New Rural Cooperative Medical Scheme(NRCMS)			
	Elasticity	Concentration index (CI)	Contribution to CI	Contribution to CI %
Gender, ref.: Male	0.2593	0.0047	0.0012	0.49%
Age, ref.:≤44				
45–59	0.0483	0.2499	0.0121	4.88%
≥ 60	0.1785	0.2887	0.0515	20.84%
Marital status, ref.: married	− 0.2118	0.0121	− 0.0026	−1.04%
Employment/retirement status, ref.: employment				
Retirement	0.0092	0.0293	0.0003	0.10%
Unemployment or student	−0.0753	− 0.1354	0.0102	4.12%
Educational level, ref.: Under primary school				
Junior	0.1087	0.0982	0.0107	4.32%
High school or above	0.0874	0.2742	0.0239	9.69%
Chronic, ref.: No chronic	0.2177	0.1491	0.0325	13.13%
Health status(EQ-VAS: self-reported),ref.:Excellent				
Good	0.1969	−0.0109	−0.0021	−0.87%
Medium	0.1688	−0.0559	−0.0094	−3.82%
Poor	0.0323	−0.0940	−0.0030	−1.23%
Very poor	0.0059	−0.1115	−0.0007	− 0.27%
Distance to the medical institution, ref.:< 1 km	0.0389	0.3673	0.0143	5.78%
Willingness to seek inpatient care, ref: Primary care hospital				
General hospital or TCM hospital (Traditional Chinese Medicine)	0.0343	0.5149	0.0177	7.14%
Per capita income	2.0327	0.0481	0.0978	39.54%
Total				102.8%

**Table 6:** Decomposition of concentration index of inpatient benefits among Urban Resident Basic Medical Insurance (URBMI) enrollees

	the Urban Resident Basic Medical Insurance (URBMI)			
	Elasticity	Concentration index (CI)	Contribution to CI	Contribution to CI %
Gender, ref.: Male	0.1449	0.0079	0.0011	0.71%
Age, ref.:≤44				
45–59	−0.0521	0.1768	0.0092	−5.69%
≥ 60	0.1064	0.4858	0.0517	31.95%
Marital status, ref.: married	−0.2509	− 0.0073	0.0018	1.13%
Employment/retirement status, ref.: employment				
Retirement	0.0627	0.2126	0.0133	8.24%
Unemployment or student	−0.0958	− 0.0675	0.0065	3.99%
Educational level, ref.: Under primary school				
Junior	0.1553	0.0191	0.0029	1.83%
High school or above	0.4329	0.1079	0.0467	28.87%
Chronic, ref.: No chronic	0.3029	0.0328	0.0099	6.14%
Health status(EQ-VAS: self-reported),ref.:Excellent				
Good	0.3854	0.0033	0.0013	0.79%
Medium	0.2044	0.0199	0.0041	2.51%
Poor	0.0372	−0.0853	−0.0032	−1.98%
Very poor	0.0071	−0.1047	−0.0007	− 0.46%
Distance to the medical institution, ref.:< 1 km	−0.0293	0.0279	−0.0008	− 0.51%
Willingness to seek inpatient care, ref: Primary care hospital				
General hospital or TCM hospital (Traditional Chinese Medicine)	0.2011	0.0502	0.0101	6.24%
Per capita income	0.7231	0.0257	0.0186	11.49%
Total				95.25%

**Table 7:** Decomposition of concentration index of inpatient benefits among Urban-Rural Basic Medical Insurance (URRBMI) enrollees

	the Urban Resident Basic Medical Insurance (URBMI)			
	Elasticity	Concentration index (CI)	Contribution to CI	Contribution to CI %
Gender, ref.: Male	0.0669	−0.0077	−0.0005	− 0.49%
Age, ref.:≤44				
45–59	−0.0186	0.1019	−0.0018	−1.84%
≥ 60	0.1009	0.3098	0.0313	30.29%
Marital status, ref.: married	−0.0768	0.0045	−0.0003	− 0.33%
Employment/retirement status, ref.: employment				
Retirement	−0.0079	0.1516	−0.0012	−1.16%
Unemployment or student	−0.1689	−0.0099	0.0017	1.62%
Educational level, ref.: Under primary school				
Junior	0.1178	0.0771	0.0091	8.81%
High school or above	0.1137	0.1972	0.0224	21.73%
Chronic, ref.: No chronic	0.2203	0.0862	0.0189	18.40%
Health status(EQ-VAS: self-reported),ref.: Excellent				
Good	0.2481	−0.0137	−0.0033	− 3.29%
Medium	0.0865	−0.0207	−0.0017	−1.74%
Poor	0.0303	−0.1259	−0.0038	− 3.69%
Very poor	0.0103	−0.2278	−0.0023	− 2.27%
Distance to the medical institution, ref.:< 1 km	− 0.0835	0.0041	−0.0003	−0.33%
Willingness to seek inpatient care, ref: Primary care hospital				
General hospital or TCM hospital (Traditional Chinese Medicine)	0.0383	0.3851	0.0147	14.29%
Per capita income	0.3919	0.0499	0.0196	18.95%
Total				98.95%

## Discussion

Although the preliminary framework of a unified Chinese medical urban-rural insurance scheme has been implemented, it remains of great importance to explore the benefit distribution of government healthcare subsidies throughout the medical insurance system. To the best of our knowledge, this study, which was conducted using representative Chinese household survey data, is the first to examine and compare the inequality of inpatient care benefits among enrollees of three medical insurance schemes under the integration of social health insurance for urban and rural residents.

In this study, we found that the amount of net benefits of inpatient care among URRBMI, NRCMS and URBMI enrollees ranked the greatest, the second greatest, and the least, respectively. This suggests that individuals receive more benefits under an integrated urban-rural insurance scheme. Factors associated with a net benefit of inpatient care received by rural and urban residents included gender, age, marital status, retirement, educational level, a history of chronic diseases, health status, willingness to seek inpatient care and per capita income. Females and those over 60 years of age received a significantly greater absolute amount of reimbursements for inpatient care, which is more significantly in NRCMS. These findings indicate that the elderly and females are main beneficiaries of net benefits, especially in rural areas. Divorced, single or separated individuals received significantly less reimbursements, indicating that marriage and family life are economically and spiritually positive factors in the effective utilization of available healthcare options. People with a higher education received more net benefits, in agreement with existing literature [32]. Higher education level is a marker of the ability to turn information into practical behaviors, resulting in a best use of healthcare. According to our analysis, individuals with higher incomes received a greater amount of benefits. This effect was greater among NRCMS enrollees, in agreement with some existing reports [20, 21]. Income may also be a key barrier preventing the poor from receiving net benefits of inpatient care. Policy should thus focus particularly on the poor, especially those in NRCMS to reduce negative effects of low income on net healthcare benefits. In addition, we found that sufferers of chronic diseases or those in poor health received significantly more benefits, in agreement with prior studies [20]. Individuals with a poor health status have relatively higher healthcare requirements and utilize healthcare to a greater extent, thus in turn receiving a greater amount of reimbursement. Individuals attending general or TCM hospitals for healthcare received greater benefits, possibly explained by higher fees charged by such facilities.

This study reveals a disproportionate concentration of inpatient care benefits among richer individuals and demonstrates that the rich received a greater amount of benefits than did the poor, in agreement with prior studies [21, 32]. In addition, we observed that this inequality among NRCMS enrollees was greater than that among URBMI participants, also in agreement with prior reports [33, 34]. Also, Chen Met et al. found that the inequality of distribution of government healthcare subsidies for inpatient care in urban area was smaller than that in rural area by measuring CI for inpatient care between urban and rural areas [19]. These findings indicate that there is a gap between urban and rural areas in the fairness of security in social health insurances, with the poor less likely to receive reimbursement and benefits if living in a rural area. A preceding study reported that the actual reimbursement ratio of URBMI enrollees was higher than that of NRCMS enrollees in China [35]. Meanwhile, existing research reveals that increasing the proportion of hospitalization reimbursement further promotes equality in benefits received[33]and reduce the incidence of catastrophic health expenditure [36].In our study, the CI of net benefit in URRBMI enrollees, which integrates medical insurance for urban and rural residents, was the lowest; approximately half of the value of CI in NRCMS enrollees. This finding indicates that the current reform of unifying urban-rural social health insurances contributes to a reduction in the inequality of net benefits obtained from government subsidies across different socioeconomic groups, in agreement with Mtei G et al. that the integration of health insurances schemes can improve equity of health financing reform in Tanzania [15]. Factors such as per capita income, age over 60, a history of chronic illnesses and, a high education level significantly affected net benefits received and were major positive contributors to net benefit inequality among different income populations. Some factors such as poor health status and distance to medical institutions of more than 1 kilometer had slightly negative contributions to net benefits for URBMI and URRBMI enrollees; however, distance to the medical institution was a positive contributor to benefit inequality for NRCMS enrollees. Therefore, improving benefits targeting to the poor involves not simply rearranging the public subsidies, but also addressing the constraints that prevent the poor from accessing healthcare services [37].

Policies aimed at increasing the benefit equality must address socioeconomic factors affecting healthcare outcomes. Specifically, strategies should be implemented to increase welfare subsidies and perfect social assistance schemes that improve the precision assistance for the poor and relieve the economic burden of diseases. Social health insurance programs should be further adjusted to focus on the disadvantaged, such as those over 60 years of age, enduring poor economic conditions or suffering from chronic diseases, increase reimbursement levels, and expand benefit packages. In addition, according to our findings, accelerating the integration of urban-rural medical insurance schemes should be an effective way to increase equity of benefit in both urban and rural areas. In addition, strategies preventing and controlling chronic diseases and improving health equity may be further strengthened to alleviate the inequality of benefit distribution. With regard to individuals with NRCMS, improving a two-way referral system and encouraging the rich to attend primary care hospitals for healthcare may further reduce benefit inequality. The unequal benefit package for members of different schemes and the lack of alignment between provider payment mechanisms for referral care is also the common challenge those countries where multiple health insurance funds have been established to achieve universal coverage face [38].

The findings of this study may provide evidence on increasing the benefit equality of health insurances for those countries where multiple health insurance funds have been established, especially some developing countries with a huge gap between urban and rural area. Some limitations of our study must be acknowledged. Firstly, we only adopted the Anhui province data from NHSS, potentially affecting finding universality and data representativeness. In addition, we only compared net benefit inequalities of inpatient care among different medical insurance programs but did not compare the same sample prior to and after insurance scheme reform. Furthermore, cross-sectional data did not allow for casual conclusions to be drawn. As the urban-rural insurance scheme integration progresses, further research is required to evaluate for any improvements in equality of benefit distribution.

## Conclusion

Individuals received more benefits for inpatient care under the integrated urban-rural health insurance scheme. Some socioeconomic factors such as gender, age, marital status, retirement, education level, history of chronic diseases, health status, and willingness to seek inpatient care and per capita income significantly affect net benefits received by individuals and reveal differences between health insurance schemes. Although a disproportionate concentration of net inpatient care benefits among the rich was observed, inequality of benefit distribution of URRBMI enrollees was less than that of NRCMS and URBMI participants, suggesting that the integration of urban-rural insurance schemes reduces net benefit distribution inequality. Per capita income, age over 60, a history of chronic diseases and high education level are major positive contributors to inequality. Expedited urban-rural insurance scheme integration and strategies focusing on socioeconomic factors will be effective methods for increasing fairness of health insurance benefit distribution.

## Abbreviations

CHF: The Community Health Fund
CI: Concentration index
NB: Net benefit
NHIF: The National Health Insurance Fund
NHSS: The National Health Services Survey
NRCMS: The New Rural Cooperative Medical Scheme
OOP: Out-of-pocket
TCM hospital: Traditional Chinese Medicine hospital
UEBMI: The Urban Employee Basic Medical Insurance
UHC: Universal health coverage
URBMI: The Urban Resident Basic Medical Insurance
URRBMI: The urban and rural residents’ basic medical insurance system
VAS: Visual analogue scale

## Footnotes

1 According to the Constitution of the People’s Republic of China, China’s administrative regions are divided into provincial administrative regions, county administrative regions and township administrative regions. County-level units in our study represents County Administrative Region, such as municipal district, county, autonomous county, county-level city, autonomous banner, forest region and special administrative region.
